# Enhancing Clinical Trials Through Synergistic Gamma Power Analysis

**DOI:** 10.3389/fpsyt.2020.00537

**Published:** 2020-06-10

**Authors:** Sokichi Honda, Mitsuyuki Matsumoto, Katsunori Tajinda, Takuma Mihara

**Affiliations:** ^1^Neuroscience, La Jolla Laboratory, Astellas Research Institute of America LLC, San Diego, CA, United States; ^2^Candidate Discovery Research Labs, DDR, Astellas Pharm Inc., Tsukuba, Japan

**Keywords:** electroencephalography (EEG), schizophrenia, bipolar disorder, autism spectrum disorder (ASD), evoked gamma, baseline gamma, Auditory Steady State Response (ASSR)

## Abstract

While the etiology of many neuropsychiatric disorders remains unknown, increasing evidence suggests that aberrant sensory processing plays a central role. For this class of disorders, which are characterized by affective, cognitive, and behavioral symptoms, electroencephalography remains the dominant tool for providing insight into the physiological and molecular underpinnings of the disease state and predicting the effectiveness of investigational new drugs. Within the spectrum of electrical activity present in the CNS, high-frequency oscillations in the gamma band are frequently altered in these patient populations. Measurement of gamma oscillation can be further classified into baseline and evoked, each of which offers a specific commentary on disease state. Baseline gamma analysis provides a surrogate of pharmacodynamics and predicting the time course effects of clinical candidate drugs, while alterations in evoked (time-locked) gamma power may serve as a disease biomarker and have utility in assessing patient response to new drugs. Together, these techniques offer complimentary methods of analysis for discrete realms of clinical and translational medicine. In terms of drug development, comprehensive analysis containing aspects of both baseline and evoked gamma oscillations may prove more useful in establishing better workflow and more accurate criteria for the testing of investigational new drugs.

## Introduction

Electroencephalography (EEG) indirectly measures neuronal activity within the brain. The technique is widely used in neurology, cognitive science, and psychophysiological research. EEG is an attractive tool in the field of translational science, as it is a non-invasive procedure that offers insight into the cell- and system-level changes that underlie both the normal and disordered function of the CNS. Clinically, the technique allows study of the efficacy and impact of investigational new drugs on both a systems and population level.

While a significant fraction of EEG-based research focuses on relatively low-frequency oscillations (1–30 Hz), research into higher-frequency oscillations in the gamma band (30–200 Hz) have revealed alterations that are closely linked to various neuropsychiatric disorders (1–4), including schizophrenia and bipolar disorder. Numerous studies (in both humans and animal models) suggest that changes in gamma oscillation likely reflect the activity of parvalbumin-positive GABAergic interneuron populations (2, 5). This cell population plays an important role in sensory input and processing, and is of particular interest in diseases where these processes are altered. Specifically, it is hypothesized that alterations in gamma oscillations are indicative of an imbalance in excitation/inhibition (E/I) arising from the loss of function within the previously mentioned interneuron population that ultimately manifest as affective, cognitive, and behavioral changes. Alterations in gamma oscillation have been reported in schizophrenia (6–9), autism spectrum disorder (10), fragile X syndrome (11), bipolar disorder (12), major depressive disorder (13), and epilepsy (14). For these reasons, tracking these changes remains one of the most commonly employed techniques in recent drug discovery research for neuropsychiatric disorders (1, 15).

Measurement of gamma oscillation is typically performed in one of two ways. Baseline (resting) gamma measures native gamma oscillations in the absence of external stimuli. In contrast, evoked (time-locked) gamma is passive gamma oscillation elicited by auditory or visual cues. Oscillatory activity typically evoked during sensory/cognitive processing is characterized by band-limited modulations, which are a key feature of an oscillatory process. Especially, evoked gamma power is typically observed ~60–100 ms after stimulus (1). This is not normally observed in spontaneous or baseline activity. Baseline measures are generally more prone to artefacts. While the source of these artefacts is not always clear, researchers have identified significant contributions from muscle/saccadic artefacts towards high-frequency EEG activity, the removal of which has allowed better identification of the “true” signal from brain (16–18). The frequency, duration, and magnitude of the elicited activity provide a composite measurement of the subject's response to a one-time or ongoing series of sensory input. When considered collectively, the two approaches offer a somewhat unique, albeit overlapping commentary on the functional status of sensory processing within the CNS.

In a clinical context, gamma oscillation may be used either as proof of pharmacology or as a proof-of-concept tool. With regard to the former, EEG-based measurements are typically employed to track changes in brain activity, including gamma oscillation following the addition of an investigational new drug. Tracking these changes, usually through longitudinal monitoring of baseline EEG activity, provides a functional biomarker of drug targeting and activity. Alongside other more traditional pharmacokinetic/pharmacodynamic (PK/PD) and tolerability analyses, this approach provides guidance on trial design, dosing, and predicts clinical efficacy. For these reasons, this type of evaluation is generally incorporated relatively early in the clinical process, generally in healthy subjects. In proof-of-concept applications, changes in gamma oscillation need be accompanied by similar changes in surrogate endpoint(s) that comment directly on the disease (e.g., cognitive measurement, PANSS scale changes, etc.). In addition, some regard specific alterations in gamma band activity—usually Auditory Steady State Response (ASSR) deficits—as a potential disease biomarker for some neuropsychiatric disorders, as well as a means of stratifying patients for clinical trials, though further investigation of these claims is needed.

## Gamma Oscillations in Understanding Disease Etiology

Studies of gamma band alterations have historically proven valuable in understanding the underpinnings of diseases with ostensible E/I imbalances. In animal models of neuropsychiatric disorder created by blunting NMDA signaling transduction in parvalbumin-positive interneurons baseline gamma power is increased, while evoked gamma power is decreased (5, 19). Similar results are observed in animals treated with NMDA antagonists (20, 21). This suggests two conclusions. First, the disruption in sensory input in the diseased state may be caused by a reduction in signal-to-noise ratio resulting from augmented baseline activity within interneurons. Second, NMDA-mediated signaling is directly implicated in maintaining E/I balance, as gamma alterations in animal models appear to mirror those of disease patients. While this last point is difficult to prove conclusively in patients, circumstantial evidence seems to support this working theory. For example, the addition of a GABA transporter inhibitor results in the increase in the evoked gamma power of healthy volunteers (22).

Gamma band changes have also contributed to the discovery that sensory balancing is not constrained to GABAergic neuron populations. Studies involving other neurotransmitters support the notion that the effects of E/I imbalance are more widespread than initially believed. Compounds targeting dopaminergic and norepinephrinergic neurons typically increase evoked gamma power (23, 24), indicating that these neurotransmitters can be indirectly surveilled using this measurement technique. Preclinical evidence in rodents also suggests that ASSR also changes in response to cholinergic neurotransmitter modulation (25). Based on these results, it stands to reason that ASSR could also be used a predictor for the efficacy of drugs targeting these classes of compounds.

The common role of the neural circuit mentioned above is their conserved function in sensory perception. Accordingly, imbalances in their regulation (either directly, or *via* manipulation of interneurons) could result in E/I imbalance. As one of the endophenotypes in neuropsychiatric disorders, dysfunction within these systems could manifest as false or warped sensory input in an affected individual. The monitoring of gamma oscillation as a means of commenting on this phenotype of neural function underscores the importance of this technique in dynamically characterizing the development of the disease from acute to a chronic state. Understanding the pathogenesis of etiologically challenging disorders is of critical importance in the development of next-generation neuropsychiatric drugs targeted to these pathways.

## Gamma Oscillations in Drug Development

The development of new drugs encompasses a wide variety of topics, including the pharmacological profile (PK/PD, ADME, etc.) of each new drug and the ensuing response of the patient (ranging from cell- and molecular changes to the amelioration of symptoms and changes in clinical endpoints). Regarding the former, resting gamma measurement offers direct commentary on the activity of a particular cell population, in this case, inhibitory neurons involved in sensory processing. As these populations are the target for numerous drug development efforts, measuring baseline gamma response following administration of an investigational drug provides valuable retrospective information on dosing levels and therapeutic windows, acts as a useful secondary biomarker of efficacy and receptor occupancy, and can be used to corroborate PK/PD data for new compounds. Studies using baseline gamma are technically suited to capture these types of data, and have proven successful in this endeavor (26, 27).

Despite their success in this regard, the use of baseline gamma measurement in the evaluation of new CNS drugs does not necessarily extend beyond PK/PD assessments. Although abnormal resting gamma power was also identified as being connected to neuropsychiatric disorders and their preclinical animal models (21, 28), several studies have not identified this abnormality (29, 30). While this matter is not fully settled, recent evidence suggests that many psychiatric diseases, including schizophrenia and bipolar disorder, are rooted in abnormalities in sensory processing that relate to the disruption of evoked gamma response. As such, ASSR, rather than baseline gamma, is likely to be the superior measuring stick of clinical efficacy for diseases in this category.

Subsequent results have substantiated this hypothesis. In schizophrenia patients, abnormal 40-Hz ASSR has been reported in the prodromal state and has persisted into the chronic phase of the disease (6–9, 31–37). This relationship extends to clinical trials, where the effect size of ASSR to schizophrenia is reportedly over 0.5 (35). The close association between schizophrenia and evoked gamma power suggests it should be feasible to use this alteration as a proxy for proof of pharmacology in patient populations. This possibility is further buoyed by the observation that ASSR alterations in rodent models of psychiatric disease appear primarily in the gamma band range (21).

The consistent appearance of altered evoked gamma is both widespread and in support of the interneuron-mediated E/I imbalance theory. Abnormal evoked gamma is present in genetic models with impaired NMDA receptor function in parvalbumin-positive knockout animals (5, 38), as well as NMDA receptor-antagonized models using ketamine, phencyclidine, and MK-801 (20, 21, 39–44). In addition to supporting the broader hypothesis, these data demonstrate the role of evoked gamma measurement in establishing reliable translational validity from the preclinical to clinical development.

Combining resting and evoked gamma has also been used to increase understanding of disease features in situations where either method alone would be insufficient. When multiple NMDA receptor antagonists (memantine or ketamine) were given to healthy volunteers and schizophrenia patients, ASSR was enhanced in both populations (45, 46). Although this observation seems contradictory to NMDA receptor hypothesis for schizophrenia, this effect was echoed in rats, where enhanced ASSR was correlated with moderate NMDA receptor occupancy (20). This might be explained by the persistent reduction of neuronal firing of interneurons, i.e., GABAergic neurons, in the cortex, which might directly influence the mode of action through which gamma oscillation develops in ASSR. Thus, moderate NMDA receptor blockade biases the excitatory/inhibitory balance toward increased excitability, which could yield beneficial effects on brain function, similar to the antidepressive effect of ketamine (47). However, the effect of NMDA receptor antagonists on gamma power may not be explained solely by the disinhibition of GABAergic activity, as higher exposure of ketamine (and accordingly higher receptor occupancy) reduces ASSR signaling, which may reflect a collapse of cortical neuronal synchrony (20, 21).

These seemingly paradoxical results were resolved with additional examination of baseline gamma under similar conditions, which revealed that resting gamma was increased under the influence of the NMDA receptor antagonists. This observation presented another possibility, namely, that the observed ASSR disruptions in patient populations are caused by excess augmented baseline gamma activity. As mentioned previously, calculating ASSR involves the measurement of both the response to stimuli and resting state activity. In this case, an increase in baseline gamma levels—presumably a product of the inhibition of the GABA interneurons—decreases the signal-to-noise ratio, and reduces evoked gamma in the absence of any change in peak response. Indeed, additional studies (20, 21, 48) confirmed that baseline gamma power was augmented under these conditions, regardless of receptor occupancy. However, evoked gamma enables the detection of sensory processing dysfunction between healthy subject and patient endophenotypes because baseline gamma alterations remain difficult to detect in patients.

Understanding the synergistic analysis of baseline and evoked gamma remains a particularly challenging area of EEG research, as some evidence suggests that interpretation of these results may not be as straightforward as originally assumed. Grent-'t-Jong, T., et al., for example, demonstrated that a higher dose of S-ketamine (10 mg, bolus injection) also enhanced task-related gamma power in healthy subjects, while ketamine significantly increased PANSS scores (49). This suggests a dissociation of gamma power abnormalities observed during acute NMDA receptor hypofunction and in schizophrenia. The dissociation of evoked gamma power at a higher dose of NMDA receptor antagonist in these conditions suggests the need to carefully revisit the NMDAR hypothesis, particularly for acute (e.g., ketamine-induced) NMDAR hypofunction, to create a more holistic understanding of the molecular, cellular, and system-level interplay. Similarly, the true meaning of baseline gamma power modulation needs to be more fully explored in future studies.

## Future Directions: Patient Stratification and Other Biomarkers

The repeated failures of investigational new drugs for neuropsychiatric disorders strongly suggest the need for new drug discovery approaches that target specific pathophysiological alterations shared in pre-stratified patient populations (50). Accumulated evidence demonstrates the excellent test-retest reliability of EEG measures (51–53), indicating the use of this method for identifying patient subpopulations that contain unique sensory processing deficits. A lack of patient stratification could affect the response rate among patients, reducing the overall efficacy and possibly contributing to a new drug's failure to achieve primary endpoints. Indeed, some research indicates that dopaminergic drugs show the bimodal response occurs based, at least in part, on the difference in the individual response before administration of the drug (54, 55).

The critical need for new and more useful biomarkers in neuropsychiatric disorders has also been well documented in the literature (56). The use of evoked gamma for biomarker-based stratification may be useful for a plethora of other neuropsychiatric disorders that display altered evoked gamma. This list includes schizophrenia, bipolar disorder (8, 12, 57–60), autism (61, 62), 22q11.2 deletion (63), and fragile X syndrome (11). For these diseases, incorporation of a prospective screen would aid in the selection of a more appropriate trial population and, in the case of ineffective compounds, possibly reveal a candidate's lack of efficacy at an earlier stage. It should be noted that the role(s) of gamma power abnormalities in the pathophysiology of each neuropsychiatric disorders needs to be carefully assessed.

Although the industry is still facing challenges regarding identification of potential responders in interventional trials, recent results may shed some light on new avenues of investigation into psychometric measures that identify biologically distinct subpopulations. For example, Swerdlow and Light propose treatment-sensitive individuals (i.e., responder) for cognitive training could be detected by the response of early auditory information processing (EAIP) after a single challenge dose of a pharmacologic agent (46, 64). Evoked gamma power and other measures of EIAP were enhanced with acute challenge of memantine, which is considered as retained plasticity in response to interventions. In a similar vein, Javitt et al. showed that there are (at least) two biologically distinct subgroups in schizophrenia that can be identified using tests for tone-matching and mismatch negativity (65, 66). Further, Light et al. proposed pre-dose mismatch negativity could predict response some interventions (51, 67). One recent effort to model EAIP using large patient datasets estimated that a microvolt change in the amplitude of mismatch negativity and P3a would produce substantial impact on improvement of cognition and psychosocial functioning, while impaired EAIP predicts poor functional outcomes resulting from impaired cognition and increased negative symptoms (68, 69). These findings support the rationale to identify the relevant patient subpopulations with EEG measures.

An obvious avenue to advancing patient stratification is to pair these emerging psychometric features with EEG measures to identify more biologically distinct subpopulations than previously thought possible. This work has been the focus of working groups like the Bipolar-Schizophrenia Network on Intermediate Phenotypes (B-SNIP), which focuses on studying the genetic, cognitive markers of these disorders. One of the resulting improvements in this area has been the selective pairing of investigational methods to create more precise diagnoses and improved detection (70). The earliest forms of this sort of paired analysis have already been applied to psychosis patients, and this approach, in somewhat different forms, could well be applied to other neuropsychiatric disorders.

Clinically, the lack of biomarker-based stratification and the failure to evaluate a compound's effect on appropriate biomarkers could create blind spots within clinical trials for this class of diseases. Based on the current understanding of these diseases, it is possible to argue that the selective, synergistic incorporation of gamma oscillation and other emerging biomarkers will result in improved trial design and revealed critical flaws (e.g., lack of efficacy) at an earlier stage.

## Conclusions

Changes within the gamma band have been proven to be durable biomarkers for neuropsychiatric patient populations, ones that offer commentary on both disease state and response to trial medications. While baseline gamma is an excellent tool for measuring the dynamic changes within target cell populations that occur in response to investigational drugs, the approach has specific limitations in reflecting the disease state and providing good predictive validity when it comes to identifying and testing patient populations. In contrast, evoked gamma measurements offer a potentially better patient stratification biomarker and measure of acute responsiveness to an investigational new drug, but are more difficult to employ in more complex time-course experiments. Given these strengths and limitations, the obvious course of action is the strategic pairing of these approaches to maximize their usefulness in new drug validation and clinical trial design.

The dual use of both baseline and evoked gamma measurement presents additional complexities, primarily related to the difficulty of incorporating EEG measurements in a clinical context. Perhaps, the largest of these challenges is the standardization of methods. Biomarker measurements require well-developed protocols, and these must match precisely in order to create translatable results between different clinical groups. This is doubly important for distributed clinical trials with multiple sites, as well as creating study-to-study consistency. Additionally, the use of evoked gamma measurement in clinical applications must be undertaken strategically, particularly for longer (time course) experiments conducted on neuropsychiatric patients that may be prone to seizures and/or have difficulty remaining still for extended testing. Further efforts might be needed to optimize protocols to fit the demands of clinical trials.

**Figure 1:**
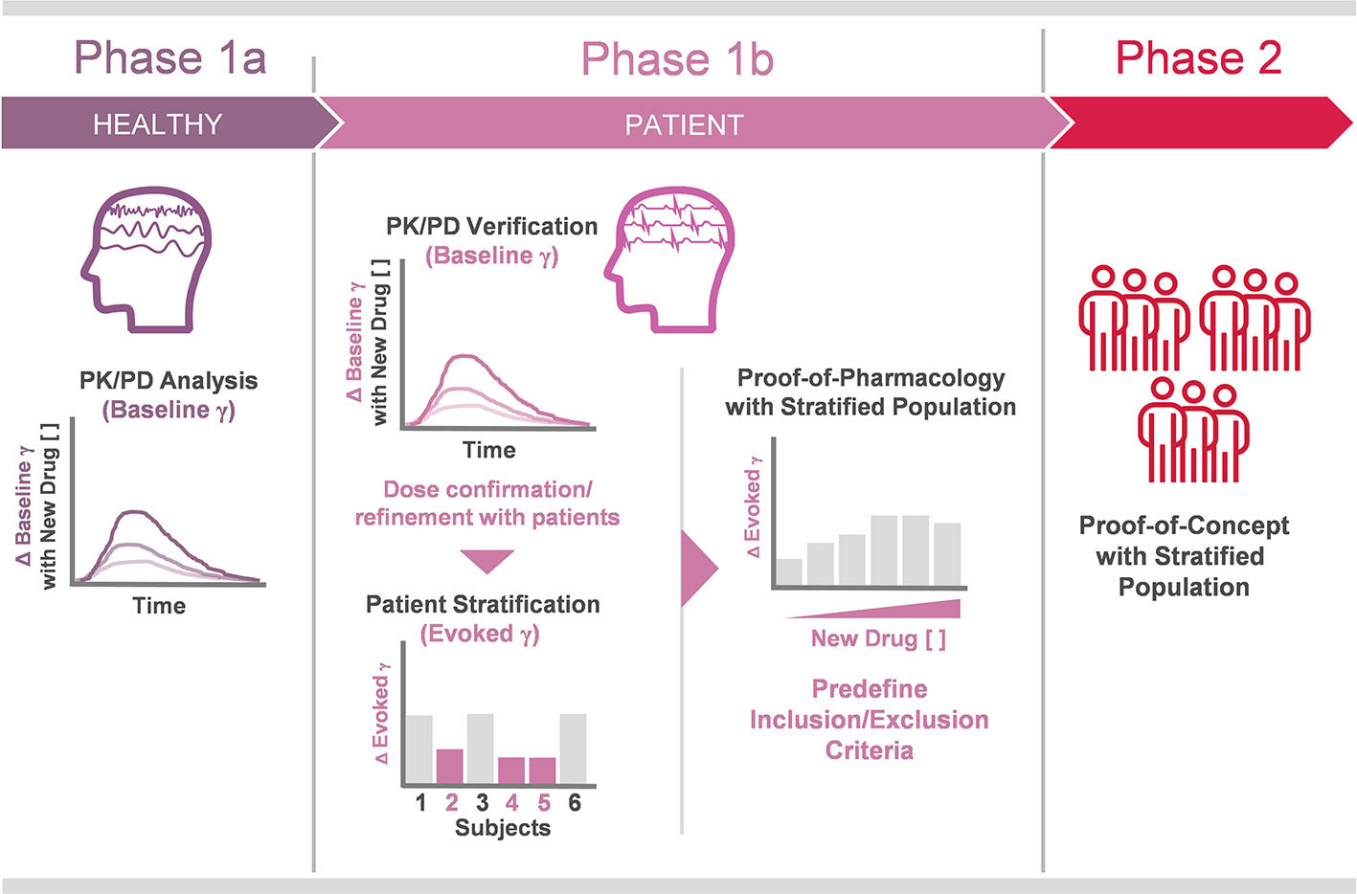
Proposed workflow of gamma oscillation analysis in future clinical trials. Phase 1a PK/PD analysis of investigational drugs targeting E/I imbalance. In the proposed trial design, baseline gamma alterations are tracked against investigational drugs concentrations in plasma, receptor occupancy, etc., in healthy volunteers. These results are confirmed in the patient population prior to stage-up. Phase 1b also includes patient population stratification, achieved in part by identification of blunted evoked gamma in ASSR testing. Phase 2 trial design is informed on dosing and time course insights gained in Phase 1a/b PK/PD testing, while candidate patient populations are defined (Phase 1b) before therapeutic response is evaluated using evoked gamma ASSR and other clinical endpoints in Phase 2.

In adapting these lessons for use in clinical trial design, several conclusions become apparent. Baseline gamma would proceed concomitantly with evoked gamma measurement in disease populations. This would inform decisions on trial design (dosing, etc.), while simultaneously stratifying a population of prospective patients for proof of concept study (**Figure 1**). After confirming the evoked gamma response in this stratified patient population, it is possible to proceed to phase two trials with increased confidence in both the design of the trial and the prospect of a more receptive patient population. As the use of baseline and evoked gamma measurements mature, we anticipate they will provide a pillar upon which to build robust translational tools and develop ever more reliable clinical protocols.

## Author Contributions

SH and TM wrote the manuscript. KT and MM participated in formative discussions and edited the manuscript.

## Conflict of Interest

All authors are employees of Astellas Pharma Inc, which is involved in the development and sale of pharmaceutical products.

## Abbreviations

Auditory Steady State Response, (ASSR); Electroencephalography, (EEG); Early Auditory Information Processing, (EAIP); Excitatory/Inhibitory, (E/I); Pharmacokinetics/Pharmacodynamics, (PK/PD).
